# Stem Cells and Cellular Origins of Breast Cancer: Updates in the Rationale, Controversies, and Therapeutic Implications

**DOI:** 10.3389/fonc.2019.00820

**Published:** 2019-08-28

**Authors:** Jiaojiao Zhou, Qishan Chen, Yiheng Zou, Huihui Chen, Lina Qi, Yiding Chen

**Affiliations:** ^1^Department of Surgical Oncology, The Second Affiliated Hospital, Zhejiang University School of Medicine, Hangzhou, China; ^2^The Key Laboratory of Cancer Prevention and Intervention, China National Ministry of Education, Hangzhou, China; ^3^Department of Cardiology, The First Affiliated Hospital, Zhejiang University School of Medicine, Hangzhou, China; ^4^Department of Clinical Medicine, Hangzhou Medical College, Hangzhou, China

**Keywords:** breast cancer, stem cell, cellular origins, therapeutic implications, heterogeneity

## Abstract

Breast cancer stem cells have been known to contribute immensely to the carcinogenesis of the breast and therapeutic resistance in the clinic. Current studies show that the population of breast cancer stem cells is heterogeneous, involving various cellular markers and regulatory signaling pathways. In addition, different subtypes of breast cancer exhibit distinct subtypes and frequencies of breast cancer stem cells. In this review, we provide an overview of the characteristics of breast cancer stem cells, including their various molecular markers, prominent regulatory signaling, and complex microenvironment. The cellular origins of breast cancer are discussed to understand the heterogeneity and diverse differentiations of stem cells. Importantly, we also outline the recent advances and controversies in the therapeutic implications of breast cancer stem cells in different subtypes of breast cancer.

## Introduction

Breast cancer is the most commonly reported type of cancer in women worldwide, and a leading cause of morbidity and mortality (1). With the morbidity and mortality associated with breast cancer, more newer therapeutic approaches are warranted. The complexity and heterogeneity of breast cancer render its treatment challenging. Gene expression profiling comprehensively demonstrated molecular portraits of breast cancer (2, 3), defining tumors as luminal-like, HER2-positive, or basal-like. Clinically, the heterogeneous breast cancer is categorized into four distinct molecular subgroups (2, 4): Luminal A and luminal B breast cancers are broadly defined as those with estrogen receptor (ERs) positive expression, which response to the antiestrogen therapy. The HER2 positive breast cancer is the subtype with high amplification of HER2 gene. The triple negative breast cancer (TNBCs, usually basal-like), lacking the expression of ER, progesterone receptor (PR), and HER2 (5), always has an increased incidence of germline BRCA1/2 mutations (6, 7). Compared to the other subtypes, TNBC is highly heterogenous, and usually have higher incidence of hematogenous metastasis. Based on a large cohort of 465 primary TNBC tumors, TNBC are now classified into transcriptome-based subtypes: luminal androgen receptor, immunomodulatory, basal-like immune-suppressed, and mesenchymal-like (8).

Two models have emerged to explain the heterogeneity of breast tumors (9). One is the clonal evolution model (10), which postulated that random mutation and clonal selection give rise to cellular heterogeneity within breast tumors. Another is the cancer stem cell model (11), which posited that the cellular diversity and tumor hierarchy are generated by the breast cancer stem cells (BCSCs). In both of the two models, tumor microenvironment plays an important role in equipping the evolution of breast cancer cells.

BCSCs (12) are defined as a small fraction of cells capable of self-renewal and propagation of the heterogeneous populations of breast cancer cells. The concept of BCSCs revealed the cellular origin, tumor maintenance, and progression of breast cancer. Clinically, BCSCs are considered to be responsible for the development of resistance to treatment and cancer relapse, through their virtue of relative resistance to radiation, cytotoxic chemotherapy and molecular targeted therapy (13).

In the present review, we provide an overview of the current advances in stem cells and the cellular origins of breast cancer. We summarized information regarding the various molecular markers of BCSCs, BCSC portraits among different subtypes of breast cancer, and associations between BCSCs, and the tumor microenvironment. Moreover, considering the contribution of BCSCs to the development of resistance to treatment and tumor recurrence, we also focused on regulatory pathways and the related therapeutic implications of BCSCs.

## Molecular Markers and Tumorigenic Features of BCSCs

BCSCs are defined as a limited group of breast cancer-initiating cells, possessing properties of self-renewal, and differentiation potential (14). In cancer research, BCSCs are usually characterized as cells that are able to form the transplantable tumors and re-establish tumor heterogeneity (15). A panel of molecular markers was used to identify the BCSCs (Table 1). Among these, CD44^+^/CD24^−/low^ (16, 17) and ALDH1^+^ (aldehyde dehydrogenase1) (18) are the most commonly used markers. Indeed, the CD44^+^/CD24^−/low^ and ALDH1^+^ phenotype cells are two distinct subpopulations of BCSCs (18). Different gene expression profiles (34) have shown that CD44^+^/CD24^−/low^ marked a mesenchymal and quiescent type of BCSCs (EMT-BCSCs, EMT: epithelial-mesenchymal transition) (35), while ALDH1^+^ labeled an epithelial and proliferative type of BCSCs (MET-BCSCs, MET: mesenchymal-epithelial transition). According to the fostered concept of transient EMT-MET switches in metastatic tumor cells (36), these two subgroups of BCSCs can be accepted as two dynamic states of BCSCs. It has been recently demonstrated that the transition between these two states of BCSCs (EMT and MET-BCSCs) can be reversely regulated by cytokine signaling such as Id1 (inhibitor of DNA binding 1) (37).

**Table 1:** Markers used to identify breast cancer stem cells, derived from breast cancer cell lines, transgenic mouse models, and patient-derived tumors.

**BCSC markers**	**Study (year)**	**Annotations of markers**	**Mouse model or cell line used for the BCSC enrichment**
**MOST COMMONLY USED MARKERS**			
CD44^+^/CD24^−/lo^	Al-Hajj et al. (16) Shipitsin et al. (17)	CD44: a cell-surface glycoprotein, interacts with ligands such as osteopontin, collagens, and matrix metalloproteinases, usually presents in progenitor cells	Patient derived xenograft tumors (malignant pleural effusion; primary tumor specimen)
ALDH1^+^	Ginestier et al. (18)	ALDH1: aldehyde dehydrogenase1, a detoxifying enzyme for the oxidation of intracellular aldehydes, functions in early differentiation of stem cells through its role in oxidizing retinol to retinoic acid	Patient-derived xenograft tumors (breast tumor specimen)
**MARKERS DERIVED FROM THE TRANSGENIC MOUSE MODEL**			
CD133^+^	Wright et al. (19)	CD133: a transmembrane glycoprotein, prominin 1, functions in maintaining stem cell properties by suppressing differentiation.	Brca1-deficient mouse (Brca1^Δ11^p53^+/−^)
CD24^+^ Thy1^+^	Cho et al. (20)	Thy1: a cell-surface antigen, also known as CD90, mediates the cell adhesion, and communication of cancer stem cells.	MMTV-Wnt-1 mouse
CD29^lo^CD24^+^ CD61^+^	Vaillant et al. (21)	CD61: β3-integrin, expressed in luminal progenitor cells, a prognostic indicator in breast cancer	MMTV-Wnt-1 and p53^+/−^ mouse
Sca1^+^	Grange et al. (22)	Sca1: stem cell antigen, also known as Ly6a, expressed in mammary gland progenitors	BALB-neuT mouse
CD24^+^CD29^+^/CD49f^+^	Vassilopoulos et al. (23)	CD29 and CD49f: β1-integrin and α6-integrin, also expressed in normal mammary stem cells	Brca1-mutant mouse (Brca1^Co/Co^p53^+/−^; MMTV-Cre)
**MARKERS DERIVED FROM THE CELL LINES**			
MUC1^+^	Engelmann et al. (24)	MUC1: a transmembrane glycoprotein, mucin1, a well-known tumor antigen of breast cancer also known as CA153	MCF-7 SP (CD44^+^/CD24^−/low^)cell line
Procr^+^/ESA^+^	Hwang-Verslues et al. (25)	Procr: protein C receptor, a known marker of hematopoietic, neural, and embryonic stem cells. ESA: epithelial specific antigen, expressed in epithelial cells	MDA-MB-231, MDA-MB-361 cell line
CD49f^+^/DLL1^hi^/DNER^hi^	Pece et al. (26)	DLL1: a member of the delta/serrate/jagged family involved in cell-to-cell communication DNER: delta/notch- like EGF repeat containing	Cells from breast tumors(well-differentiated/G3 or poorly-differentiated breast cancer)
GD2^+^	Battula et al. (27)	Ganglioside GD2: a glycosphingolipid, highly expressed on bone marrow- derived mesenchymal stem cells	HMLER, MDA-MB-231 cell lines
CD44^+^/CD24^−/lo^/ANTXR1^+^	Chen et al. (28)	ANTXR1: ANTXR cell adhesion molecule 1, can interact with LRP6 and VEGFR and modulate Wnt and VEGF signaling	MCF-10A, TMD-231 cell lines
ABCG2^+^	Leccia et al. (29)	ABCG2: a transmembrane transporter, ATP- binding cassette subfamily G member 2, expressed in normal, or cancer stem cells	HCC1937 cell line (BRCA-1 mutated basal- like cell line)
Lgr5^hi^	Yang et al. (30)	Lgr5: a Wnt signaling target gene, a stem cell marker overexpressed in breast cancer	MCF-7, MDA-MB-231 cell line
CD44^+^CD24-^/lo^SSEA-3^+^ or ESA^hi^PROCR^hi^SSEA-3^+^	Cheung et al. (31)	SSEA-3: stage-specific embryonic antigen-3, the globo-series glycan	MCF-7, MDA-MB-231 cell line
Nectin-4^+^	Siddharth et al. (32)	Nectin-4: a family of immunoglobulin-like cell adhesion molecules crucial for the formation and maintenance of Cadherin-based adherens and Claudin-based tight junctions	MDA-MB-231 cell line
CD70^+^	Liu et al. (33)	CD70: a type II transmembrane protein, a member of the TNF receptor superfamily	231-LM2 cell line (a highly lung-metastatic sub-line derived from MDA-MB-231), CN34-LM1 cell line (a lung-metastatic derivative of another breast cancer cell line CN34)

Numerous other BCSCs markers were identified in different mouse models and breast cancer cell lines (Table 1). Wright et al. (19) reported that BRCA1-deficient mouse mammary tumors—which mimic the BRCA1-associated breast cancer—harbor heterogeneous BCSCs subpopulations including CD133^+^ (prominin1) and CD44^+^/CD24^−^ stem cells. Vassilopoulos et al. (23) found that CD24^+^CD29^+^/CD49f^+^ enriched BCSC population in BRCA1-mutant mice displayed enhanced metastatic potential. BCSCs in breast tumors of MMTV-Wnt-1 and p53^+/−^mice were identified by the marker of Thy1 (cell surface antigen also known as CD90) (20) and CD61 (21). In addition, Sca-1^+^ (stem cell antigen 1) marked a BCSC subpopulation in the BALB-neuT mouse model (22).

Various BCSC markers were also identified from breast cancer cell lines such as MUC1 (also known as CA153) (24), Procr^+^/ESA^+^ (epithelial specific antigen) (25), DLL1^+^/DNER^+^ (delta-like canonical Notch ligand1/delta/notch-like EGF repeat containing) (26), GD2 (27), ANTXR1 (ANTXR cell adhesion molecule 1) (28), ABCG2 (ATP-binding cassette subfamily G member 2) (29), Lgr5 (leucine rich repeat containing G protein-coupled receptor 5) (30), SSEA-3 (stage-specific embryonic antigen-3) (31), Nectin-4 (nectin cell adhesion molecule 4) (32), and CD70 (33) (Table 1).

Considering the heterogeneity of breast cancer, the variety of BCSCs markers observed in different studies may be attributed to different levels of breast cancer hierarchy. Moreover, it may be explained as the results of dynamic states of BCSCs, regulated by the microenvironment. Further *in vivo* and patient-derived xenograft studies are required for the definitive identification of BCSCs.

## Signaling Pathways Regulating BCSCs

Given their self-renewal and tumor-initiating properties, BCSCs have emerged as the “ringleader” for the development of therapeutic resistance in breast cancer (38). Therefore, BCSC-related therapeutic options, such as targeting the main regulatory signaling pathways in BCSCs, have recently been developed for the treatment of breast cancer (Table 2), especially in case with therapeutic resistance.

**Table 2:** Breast cancer stem cells-targeted therapies in the treatment of breast cancer and their potential mechanism of breast cancer stem cell eradication.

**Therapeutic targeting mechanism**	**Drug class**	**Drug**	**Functional mechanism of BCSC eradication**
Wnt pathway	Frizzled 7 inhibitors	Vantictumab	Inhibiting Wnt signaling by blocking the Wnt receptor Feizzled 7
Hedgehog pathway	SMO(Smoothened) inhibitor	Vismodegib Sonidegib	Inhibiting Hedgehog signaling by blocking the Smoothened, leading to the inactivation of the Gli, which regulates the tumor-mediating genes
	GLI1/2 inhibitors	GANT61	Inhibiting Hedgehog signaling by blocking the Gli1 and Gli2, which regulates the tumor-mediating genes
Notch pathway	γ-secretase inhibitors	MK-0752 PF-03084014 RO-4929097	Inhibiting Notch signaling by stopping the Notch intracellular domain into the nucleus
DNA-repair deficiency	PARP inhibitors	Olaparib	Inhibiting the DNA repair of the cancer cells by trapping the PARPs
Cell cycle	CDK inhibitors	Palbocilib	Impeding cancer cell proliferation by inhibiting the CDKs (such as CDK4/6 causing G1 arrest)
PI3K/Akt/mTOR	mTOR inhibitors	Everolimus	Killing cancer cells by targeting the mTOR in PI3K/Akt/mTOR pathway, which is pivotal in cancer cell protein synthesis, proliferation, invasion, and survival
PI3K	PI3K inhibitor	GDC-0941	GDC-0941 is a pan-PI3K inhibitor that suppresses BCSCs in combination with EGFR/Notch bispecific antibody PTG12
		Alpelisib	Alpelisib is a PI3K inhibitor that functions in PIK3CA-althered luminal breast cancer, including the endocrine therapy-resistant cases
HER-2	HER-2 inhibitor	Trastuzumab pertuzumab lapatinib TDM-1	May inhibit the HER-2 related BCSC-activating pathways
AR	AR inhibitor	Enzalutamide	Targeting BCSCs through androgen signaling pathway
PR	Progestrone antagonist	Mifepristone	Suppressing BCSCs by down-regulating KLF5 expression through inducing miR-153 expression
Aldehyde dehydrogenase	Antialcoholism drug	Disulfiram	Targeting BCSCs and reversing the pan-chemoresistance in breast cancer cells
Anti-hyperglycemic	Diabetes mellitus drug	Metformin	Decreasing BCSCs through degrading KLF5 and its downstream target genes including Nanog and FGF-BP1
Isothiocyanate	Cancer prevention agent	Sulforaphane	Eliminating BCSCs by inhibiting NF-kB p65 subunit translocation and downregulating p52 and consequent downstream transcriptional activity
HIF-1α	HIF-1α vaccination	HIF-1a–specific IgG	Immunization against HIF-1α inhibits the tumor growth in TNBC models of C3(1)Tag mice and decrease SCa-1 marked BCSCs.
HSP90	C-terminal HSP90 inhibitor	L80	Inhibiting AKT/MEK/ERK/JAK2/STAT3 signaling and suppressing CD44+/CD24-BCSCs
Nanomedicine	nanoparticles	Gd@C82(OH)22	Blocking EMT transition with resultant efficient elimination of BCSCs through inhibiting HIF-1α and TGF-βactivities

**Figure 1:**
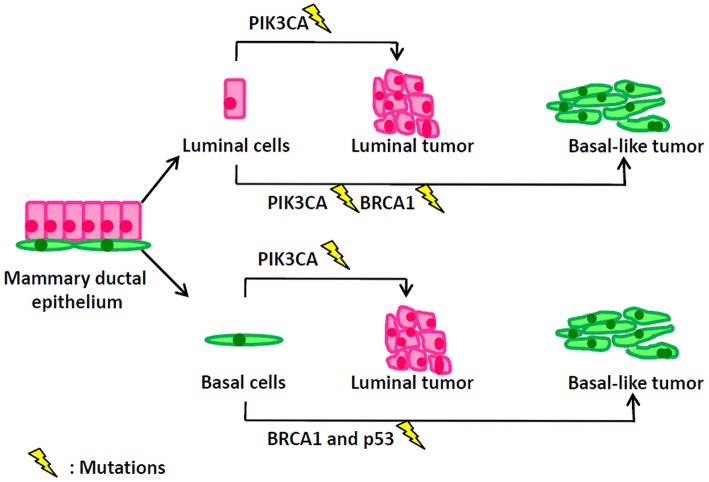
Mutation events in different types of mammary cells lead to distinct types of breast tumors.

The major signaling pathways (39) regulating BCSCs include Wnt (40), Notch (41), and Hedgehog (42) (Figure 1). Inhibitors blocking these signaling were developed as the BCSC-targeting therapies (Figure 1). (1) Increased activation of the Wnt pathway is usually found in BCSCs, leading to the nuclear translocation of cytosolic β-catenin to activate the Wnt-targeted genes, by binding the TCF/LEF (T cell factor/lymphoid enhancing factor) family. Recently, the Wnt heterodimer receptor (i.e., FZD7 and LRP6: frizzled-7 and lipoprotein receptor related protein-6) was found to be up-regulated in the TNBCs. Knockdown of this receptor suppressed tumor growth (43). Targeted medicines for the inhibition of the FZD7 receptor (i.e., Vantictumab) have been developed (44, 45). (2) The Hedgehog pathway is also crucial in BCSCs. Binding of Hedgehog to the Patched alleviates its inhibition of Smoothened. The activated Smoothened subsequently releases Gli to regulate Hedgehog target genes (46). Higher expression of Smoothened was found in a BCSC subpopulation (CD44^+^/CD24^−^ cells) (47) and the inhibitor of Smoothened (i.e., Vismodegib, Sonidegib) has already been investigated in clinical trials (48, 49). Besides, the anti-cancer stem cell activities of Gli1/2 inhibitor (GANT61) were also proved in TNBCs (50). (3) The Notch pathway serves as a key signaling cascade involved in the maintenance of BCSC phenotype. Notch ligands (e.g., Delta-like 1, 3, 4, and Jagged 1, 2) binding to Notch receptors result in the release of Notch intracellular domain (NCID) (41). With the help of γ-secretase, NCID translocates into the nucleus to activate several downstream effectors (41). McGowan et al. (51) demonstrated that CD44^hi^/CD24^lo^BCSCs contributed to the brain metastases of breast cancer, partially arisen from increased Notch activity. Clinically, drugs (e.g., MK-0752, RO-4929097, and PF-03084010) (52) targeting the γ-secretase—through the mechanism of curbing Notch signaling by stopping the NCID into the nucleus—are currently underway for the treatment of breast cancer.

## MicroRNAs Regulating BCSCs

Epigenetic regulation of microRNAs is important in BCSCs (53). MiR-200 family was proved to be a critical regulator for BCSCs growth and function. MiR-200c can strongly suppress the clonogenicity of BCSCs by targeting BMI1 (BMI1 proto-oncogene, polycomb ring finger) (54). MiR-200b directly acts on Suz12 (SUZ12 polycomb repressive complex 2 subunit), and the miR-200b-Suz12-cadherin pathway functions on BCSC growth (55). MicroRNA-200c/141 was found to regulate the heterogeneity of the BCSCs and promote the EMT-like BCSC generation, by targeting HIPK1 (homeodomain-interacting protein kinase 1)/β-catenin axis (56). Let-7 was found to regulate BCSCs by silencing H-RAS (HRas proto-oncogene, GTPase) and HMGA2 (high mobility group AT-hook 2), resulting in reduction of self-renewal and enhancement of differentiation of BCSCs (57). Similar to let-7, reduction of microRNA-30 in BCSCs contributes to the maintenance of self-renewal capacity in BCSCs, by targeting UBC9 (ubiquitin-conjugating enzyme 9) and ITGB3 (integrin b3) (58). In addition, in BCSCs, miR-600 acts as a bimodal switch that regulates WNT Signaling through SCD1 (stearoyl desaturase 1), balancing the self-renewal, and differentiation of BCSCs (59). More microRNAs and their clusters need to be investigated in BCSCs, especially for their roles in stem cell maintenance of self-renewal, differentiation, and EMT transition. Clarifying the microRNA regulation of BCSCs can further advance or understanding of the roles of BCSCs in breast cancer progression.

## BCSCs and the Tumor Microenvironment

BCSCs are located in the tumor microenvironment, which is also called as BCSC “niche.” The BCSC niche plays a vital role in sustaining the function of BCSCs. It is a complex network, containing stroma cells [such as mesenchymal stem cells (MSCs), cancer associated fibroblasts (CAFs), adipocytes, endothelial cells, and immune cells], extracellular matrix (ECM) components, cytokines, growth factors, and physical factors (such as hypoxia).

MSCs are the multipotent mesenchymal stomal cells which can be recruited from the bone marrow or normal breast stroma. It has been shown that bone marrow-derived MSCs can expand the BCSC population through cytokine loops involving IL6 (interleukin 6) and CXCL7 (pro-platelet basic protein) (60). It was also found that MSCs can propagate traits of BCSCs by promoting the contact-dependent upregulation of microRNA-199a and subsequent repression of FOXP2 (forkhead-box P2) (61). CAFs are activated fibroblasts in the tumor-hosting niche, which can promote cancer progression, especially that of breast cancer. It was reported that CAFs can regulate BCSCs through factors such as CCL2 (monocyte chemotactic protein-1) (62), IL-6 and IL-8 (63). Moreover, autophagic CAFs can promote stemness of luminal breast cancer cells by releasing HMGB1 (high-mobility group box 1) (64). Conversely, BCSCs can also regulate CAFs via signaling such as Hedgehog, in which CAFs subsequently promote the expansion and self-renewal of BCSCs (49, 65). Immune cells, especially the tumor-associated macrophages (TAMs) –are closely associated with tumor propagation. Tumor cells produce M-CSF (macrophage colony-stimulating factor) to expand TAMs, while TAMs produce TNFα and TGFβ to facilitate CSCs (66). In breast cancer, TAMs can promote BCSCs through a paracrine EGFR/Stat3/Sox-2 signaling pathway (67), while upregulation of HAS2 (hyaluronan synthase 2) in CD44^+^/CD24^−^/ESA^+^ BCSCs can enhance the interaction between BCSCs and TAMs, resulting in the BCSC growth (68). Other immune cells, such as tumor-infiltrating lymphocytes (TILs, including CD4^+^, CD8^+^, and FOXP3^+^ TILs) are also closely correlated with BCSC phenotypes, therapeutic response, and prognosis in breast cancer (69). Recent research by machine learning identified that immune microenvironment content and PD-L1 levels associated with the stemness of breast cancer (70). Adipocytes constitute a major component of the breast stroma which provide pro-tumorigenic signals in breast cancer (71). In the Goto et al. study (72)—using a PDX (Patient derived xenograft) model—it was found that adipose tissue secreted adipsin to enhance BCSC properties in breast cancer. Endothelial cells are necessary in tumor angiogenesis, which is important for nutrient and oxygen supply in the tumor microenvironment. Independently of their vascular functions, Ghiabi et al. (73) found that endothelial cells can enrich the CD44^+^/CD24^−^ stem population in breast cancer.

The ECM is a three-dimensional network of extracellular macromolecules that confines tumor cells in the microenvironment, thereby sustaining tissue homeostasis. It was illustrated that stiffness of the ECM influenced breast cancer cells through the YAP/TAZ (Yes-associated protein/Transcriptional coactivator with PDZ-binding motif) (74). Hpoxia is very important in sustaining the quiescent state of stem cells by activating the hypoxia-inducible factor (HIF). In breast cancer, hypoxia induces the BCSC phenotype through HIF-dependent and ALKBH5-mediated m(6)-demethylation of NANOG mRNA (75).

## Cellular Origins of Breast Cancer

The concept of “cellular origins” is closely associated to—but quite distinct—from the notion of “cancer stem cells” (76). The BCSC concept highlights a tiny population of the breast tumor-initiating cells that can maintain tumorigenesis and seed metastases, while the notion of cellular origins emphasizes the original normal cell types in the breast which generate a full-blown tumor. The diversity of the phenotypes displayed by breast cancer cells stimulates interests in investigating the cellular origins of this disease.

Historically, in breast cancer, the name “luminal” of luminal-A/B subtypes and the name “basal-like” of basal-like subtype are derived from similarities in transcriptomes between breast tumors and the corresponding normal mammary luminal or basal epithelium. However, the real cellular origins of luminal and basal-like breast cancer are greatly different from their naming rules. Oncogenic events in different types of mammary cells lead to distinct types of breast tumors (Figure 1). PIK3CA (α-catalytic subunit of PI3K) mutations occur in 30% of breast cancers, including both luminal and basal-like tumors. However, Meyer et al. (77) reported that the mutant PIK3CA in mammary luminal progenitors generated heterogeneous tumors of both luminal and basal differentiation. Van Keymeulen et al. (78) also found that expression of the PI3KCA mutant in luminal cells—marked by CK8—induced the luminal or basal-like breast tumors, while its expression in basal cells—marked by CK5—gave rise to the luminal tumors. BRCA1 basal-like breast cancers may originate from basal stem cells. However, interestingly, Molyneux et al. (79) demonstrated that deletion of BRCA1 in mammary luminal epithelial cells—targeted by Blg—can generate basal-like breast tumors, phenocopying the human BRCA1-associated breast cancers, while the deficiency of BRCA1 in basal cells– targeted by CK14^–^ can only generate the malignant adenomyoepitheliomas which are rare in human BRCA1-associated breast cancer. Furthermore, Tao et al. (80) depicted that the CK8^+^ luminal cells carrying the Etv6-NTRK3 fusion oncogene can induce the heterogeneous tumors with the expression of luminal and basal markers. Compelling evidence showed that luminal progenitors can serve as the cellular origins of both luminal- and basal-like human breast cancers, while the distinct genetic mutations—occurring in the transformation of luminal progenitors—are probably determinant of the eventual luminal-like or basal-like tumor phenotypes (81). Genetic sequencing results have illustrated different mutation profiles between luminal-like and basal-like tumors. The luminal-like tumors present distinct mutations, such as PIK3CA (82, 83), GATA3 (84, 85) and FOXA1 (84), while the basal-like tumors exhibit high rates of p53 and BRCA1 mutations. Using conditional mouse models, Liu et al. showed that somatic loss of both BRCA1 and p53 did result in the development of basal-like breast cancer (86).

The cellular origins of the rare type of breast cancers, such as metaplastic carcinoma should also be mentioned. Molecularly, this metaplastic subtype is similar to claudin-low breast cancer. Keller et al. (87) found that the transformation of CD10^+^ basal cells gave rise to rare metaplastic tumors. McCarthy et al. (88) demonstrated that these metaplastic tumors frequently harbored the p53 mutation and aberrant BRCA1 expression.

## BCSCs Among Different Subtypes of Breast Cancer and Their Therapeutic Implications

Different subtypes of breast cancer exhibit different abundances of BCSCs, as well as varying proportions of epithelial or mesenchymal BCSC subtypes. It is commonly recognized that BCSCs are much more enriched in the TNBCs and HER2 subtypes vs. luminal breast cancer. In the model illustrated by Brooks et al. (9), claudin-low TNBCs are characterized by a high proportion of mesenchymal BCSCs with CD44^+^/CD24^−/lo^ expression, while basal-like TNBCs contain a subcomponent of mesenchymal BCSCs and a higher proportion of epithelial ALDH1^+^ BCSCs. Liu et al. (89) compared the transcriptional profiles of epithelial or mesenchymal BCSC subtypes in TNBCs and found that the bi-BCSC subgroup (i.e., ALDH1^+^ and CD44^+^/CD24^−/lo^) was highly purified, with expression of prognostic genes such as P4HA2, PTGR1 and RAB40B.

**Figure 2:**
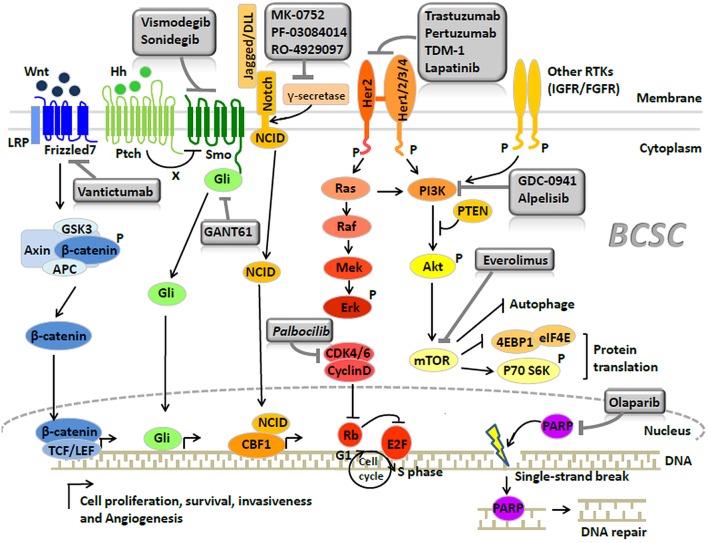
Signaling pathways and breast cancer stem cell-targeted agents in breast cancer (Copyright: Springer Nature 2014 and 2016).

TNBCs harbor the highest proportion of BCSCs compared with other subtypes, contributing to the poor prognosis associated with this subtype (90). Currently, the only established treatment against TNBCs is the cytotoxic chemotherapy; however a considerable number of patients develop resistance (5, 91). Recently, a PARP (poly-ADP-polymerase) inhibitor—through the underlying mechanism of inhibiting DNA repair, has demonstrated good efficacy against BRCA1-associated breast cancer, which usually refers to TNBCs. In breast cancer cell lines, PARP inhibitor (Olaparib) can significantly decrease the proportion of BCSCs with CD44^+^/CD24^−/low^/ESA^+^ cell surface marker, indicating the potential activity of PARP inhibitor in anticancer stem cells (92) (Figure 2, Table 2). However, Liu et al. (93) recently reported that there were some BCSCs in BRCA1-mutant TNBCs which were relatively resistant to PARP inhibitor (Olaparib), and reduction of RAD51 can sensitize these BCSCs to Olaparib treatment. Clinical benefits from the EGFR inhibitor (94) and a pan-PI3K inhibitor (95) have been reported in TNBC patients, while Fu et al. (96) further reported that EGFR/Notch bispecific antibody PTG12 in combination with pan-PI3K inhibitor GDC-0941 exerted a stronger antitumor effect in TNBC tumors by inhibiting the stem cell–like subpopulation and reducing tumor-initiating cell frequency (Figure 2, Table 2). Up to half of all TNBCs express androgen receptor (AR) (97). AR-targeted therapies (Enzalutamide) can decrease a BCSC-like population in TNBC cell lines (98), indicating Enzalutamide may enhance the efficacy of chemotherapy by targeting a BCSC like cell population (Figure 2, Table 2). In TNBCs, hypoxia-driven BCSCs abated the effectiveness of paclitaxel-based chemotherapy and antiangiogenic agents (e.g., VEGF inhibitors bevacizumab), through HIF-1α (hypoxia-inducible factors 1α). In pre-clinical models, the co-administration of HIF-1α inhibitors was able to overcome BCSC-related resistance (99, 100). HIF-1α vaccination can also inhibit tumor growth in TNBC models of C3(1)Tag mice and decrease SCa-1 marked BCSCs (101) (Table 2). Recently, Cho et al. (102) reported that L80, which is the C-ring truncated deguelin derivative as a C-terminal HSP90 inhibitor, can effectively target BCSC-like trait in TNBCs, together with obvious reduction in CD44^+^/CD24^−^ cancer cell population, ALDH1 activity and mammosphere forming-ability (Table 2).

Some drugs were repurposed as the BCSC inhibitors in TNBCs (Table 2). Disulfiram, an antialcoholism drug, was found to target BCSCs and reverse the acquired pan-chemoresistance in TNBC cell lines (103) (Table 2). Mifepristone, a progesterone antagonist for abortion, was reported to suppress BCSCs in TNBC tumors by down-regulating Krüppel-like factor 5 (KLF5) expression, which is a stem cell transcription factor over-expressed in basal type TNBC (104) (Table 2). Metformin, a first-line drug for type 2 diabetes mellitus, was found by the same group that can decrease the BCSC population in TNBC, also by targeting KLF5 for degradation (105) (Table 2). Besides, sulforaphane, a cancer prevention agent, was found to preferentially eliminate BCSCs by inhibiting NF-kB p65 subunit translocation and downregulating p52 (106) (Table 2). And Sulforaphane can also reverse taxane-induced ALDH^+^ BCSC enrichment (106). Interestingly, novel medicines such as nanomedicines, Gd@C_82_(OH)_22_ nanoparticles, were found possess intrinsic inhibitory activity against BCSCs in claudin-low TNBC cell lines (107).

In Brook's model, HER2 breast cancer is characterized by a high proportion of epithelial ALDH1^+^ BCSCs (9). HER2 is a crucial regulator in BCSCs (108). Recent studies indicated that the regulation of BCSCs by HER2 was not observed only in HER2 breast cancer but extended to all the subtypes of breast cancer (109). To some extent, the remarkable clinical efficacy of HER-2 inhibitors (e.g., trastuzumab, pertuzumab, lapatinib, and TDM-1) was attributed to target BCSCs (109) (Figure 2, Table 2). Nevertheless, a proportion of HER-2 amplified breast cancers continue to eventually develop drug resistance, probably due to the PTEN (phosphatase and tensin homolog) loss or activation of PIK3CA mutation (110). A recent study performed by Sun et al. (111) showed that MEOX1 (mesenchyme homeobox 1) may be a novel target in BCSCs of PTEN-deficient trastuzumab-resistant breast cancers. However, the precise role of BCSCs in the development of resistance to HER-2 inhibitors remains elusive. Thus, further investigation is required to elucidate this process.

Luminal A breast cancer had the lowest proportion of BCSCs among all the breast cancer subtypes, presenting the best prognosis (9). Luminal B breast cancer displayed a certain proportion of BCSCs, which was lower than those observed in TNBC or HER2 breast cancer. A proportion of luminal-B patients have poor prognosis, supposed to be the result of containing a proportion of the BCSCs (112). The presence of the BCSCs is also regarded as the main cause of resistance to hormonal therapy in luminal breast cancer (113, 114), which can be regulated by the CyclinD-CDK4/6 (cyclin-dependent kinase 4/6) complex (115, 116) and mTOR signaling (117) (Figure 2, Table 2). Use of CDK4/6 (i.e., palbocilib) (118) or mTOR inhibitor (i.e., everolimus) (119) significantly improves the survival of patients who develop endocrine resistance. Recently, the PI3K inhibitor (i.e., alpelisib) was also proved to have great clinical activity in PIK3CA-altered luminal breast cancers, including the endocrine therapy-resistant cases (120).

As described above, BCSCs are of great therapeutic relevance, particularly in overcoming resistance to treatment. Although pre-clinical models and clinical trials yielded promising results in targeting BCSCs, the efficacy and safety of BCSC-targeted therapy requires further evaluation. BCSCs share the similar markers and signaling pathways with mammary stem cells. Currently, it is unclear whether BCSC-targeting agents may also target normal mammary stem cells, resulting in severe treatment-related side effects. Besides, the clinical trial data have shown that addition of BCSCs- targeting agents to chemotherapy or hormonal therapy improved treatment efficacy. However, the combination of BCSC-targeting agents and different conventional therapies requires further investigation.

## Conclusions and Perspectives

Impressive advances have been witnessed in understanding the carcinogenesis of breast cancer, in which BCSCs hypothesis provided very important models. BCSC-targeted therapy may eradicate the cancer stem cells, which are regarded as the “the seed of tumor.” Therefore, indubitably, the clinical relevance of BCSCs is a primary concern. However, the BCSC population is heterogeneous, involving various cellular markers and regulatory signaling pathways. In addition, the BCSC microenvironment is complex. These facts render BCSC-targeted therapy difficult. Also, different subtypes of breast cancer exhibit distinct BCSC subtypes and abundances. This evidence calls for appropriate BCSC- targeted regimens against different subtypes of breast cancer. Moreover, considering that BCSCs only constitute a small fraction of the tumor cells, the classic clinical endpoints of tumor shrinkage may be inappropriate to assess the efficacy of BCSC-targeted therapy. Recent studies have indicated that the detection of BCSCs markers in tumor biopsies or even in circulating tumor cells may be a favorable option (121, 122).

In conclusion, despite challenges ahead, the comprehensive understandings of stem cells and the cellular origins of breast cancer have assisted and will continue to assist treating physicians in ultimately overcoming the stubborn aspects of breast cancer.

## Author Contributions

JZ, YZ, and QC mainly drafted the manuscript. JZ and YC supervised and edited the manuscript. YZ, HC, and LQ participated in drawing the pictures. QC edited the language of the manuscript. All authors read and approved the final manuscript.

### Conflict of Interest Statement

The authors declare that the research was conducted in the absence of any commercial or financial relationships that could be construed as a potential conflict of interest.

## Abbreviations

BCSCs: Breast cancer stem cells
CD44: CD44 molecule
CD24: heat-stable antigen
ALDH1: Aldehyde dehydrogenase1
EMT: epithelial-mesenchymal transition
MET: mesenchymal-epithelial transition
Id1: inhibitor of DNA binding 1
CD133: prominin1
CD29: β1-integrin
CD49f: α6-integrin
Thy1: Thy-1 cell surface antigen, CD90
CD61: 1, Integrin subunit beta 3, ITGB3
Sca-1: Stem cell antigen 1
MUC1: also known as CA153
ESA: Epithelial specific antigen
DLL1: Delta like canonical Notch ligand1
DNER: Delta/notch like EGF repeat containing
GD2: Ganglioside GD2
ANTXR1: ANTXR cell adhesion molecule 1
ABCG2: ATP binding cassette subfamily G member 2
Lgr5: leucine rich repeat containing G protein-coupled receptor 5
SSEA-3: stage-specific embryonic antigen-3
Nectin-4: nectin cell adhesion molecule 4
CD70: CD70 molecule
TCF/LEF: T cell factor/lymphoid enhancing factor
FZD7: Frizzled-7
LRP6: Lipoprotein receptor related protein-6
MaSCs: Mammary stem cells
NCID: Notch intracellular domain
BMI1: BMI1 proto-oncogene, polycomb ring finger
Suz12: SUZ12 polycomb repressive complex 2 subunit
HIPK1: Homeodomain-interacting protein kinase 1
H-RAS: HRas proto-oncogene, GTPase
HMGA2: High mobility group AT-hook 2
UBC9: Ubiquitin-conjugating enzyme 9
ITGB3: Integrin b3
SCD1: Stearoyl desaturase 1
MSCs: Mesenchymal stem cells
CAFs: Cancer associated fibroblasts
ECM: Extracellular matrix
IL6: interleukin 6
CXCL7: pro-platelet basic protein
FOXP2: forkhead-box P2
CCL2: Monocyte chemotactic protein-1
HMGB1: High-mobility group box 1
TAMs: Tumor associated macrophages
M-CSF: Macrophage colony-stimulating factor
TNFα: Tumor necrosis factor alpha
TGFβ: Transforming growth factor beta
CSCs: Cancer stem cells
EGFR/Stat3/Sox-2: Epidermal growth factor receptor/ signal transducer and activator of transcription 3/ SRY-box 2
HAS2: Hyaluronan synthase 2
TILs: Tumor-infiltrating lymphocytes
CD4: CD4 molecule
CD8: CD8 molecule
FOXP3: forkhead box P3
PDX: Patient derived xenograft
YAP/TAZ: Yes-associated protein/Transcriptional coactivator with PDZ-binding motif
HIF: hypoxia inducible factor
ALKBH5: AlkB homolog 5
NANOG: Nanog homeobox
PIK3CA: α-catalytic subunit of PI3K
CK8: Keratin 8
CK5: Keratin5
CK14: Keratin14
BRCA1: DNA repair associated
GATA3: GATA binding protein 3
FOXA1: Forkhead box A1
CD10: 0, Membrane metalloendopeptidase, MME
ER: Estrogen receptor
TNBC: Triple negative breast cancer
P4HA2: Prolyl 4-hydroxylase subunit alpha 2
PTGR1: Prostaglandin reductase 1
RAB40B: Member RAS oncogene family
PARP: poly-ADP-polymerase
HIF-1α: Hypoxia-inducible factors 1α
AR: androgen receptor
KLF5: Krüppel-like factor 5
CDK4/6: Cyclin-dependent kinase 4/6
PTEN: Phosphatase and tensin homolog
MEOX1: Mesenchyme homeobox 1.
